# ApoTransferrin: Dual Role on Adult Subventricular Zone-Derived Neurospheres

**DOI:** 10.1371/journal.pone.0033937

**Published:** 2012-03-30

**Authors:** Lucas Silvestroff, Paula Gabriela Franco, Juana María Pasquini

**Affiliations:** Departamento de Química Biológica e Instituto de Química y Fisicoquímica Biológica (IQUIFIB), Facultad de Farmacia y Bioquímica, Universidad de Buenos Aires (UBA), Consejo Nacional de Investigaciones Científicas y Técnicas (CONICET), Junín 956, Ciudad Autónoma de Buenos Aires (C1113AAD), Buenos Aires, Argentina; Universitätsklinikum Carl Gustav Carus an der Technischen Universität Dresden, Germany

## Abstract

Neural stem and progenitor cells (NSC/NPCs) are multipotent self-renewing cells that are able to generate neurons, astrocytes and oligodendrocytes (OLs) within the adult central nervous system. We cultured NSC/NPCs from the rat subventricular zone as neurospheres (NS) and studied apoTransferrin (aTf) effects on oligodendroglial specification and maturation. Our findings suggest that aTf acts at different stages during progression from NSC to mature oligodendrocytes. On the one hand, an early event associated with the activation of NSC/NPCs proliferation and commitment toward the oligodendroglial fate, as indicated by increased BrdU incorporation, larger neurospheres production, and higher ability to generate OL precursors (OPCs) from undifferentiated cultures. On the other hand, aTf exposure during differentiating conditions favours OL maturation from OPCs by promoting OL morphological development. This evidence supports a key role of Tf on the generation of OL from NSC/NPCs and highlights its potential in demyelinating disorder treatment.

## Introduction

Undifferentiated neural stem cells (**NSCs**) within the adult mammalian brain are one of the main targets of Multiple Sclerosis (**MS**) therapeutic strategies for myelin repair [1], [2]. These NSCs are considered a subtype of multipotent astrocytes [3], [4] that are found in the subventricular zone (**SVZ**) and hippocampus subgranular zone [5]–[7]. The adult SVZ not only contains multipotent NSCs, but neural progenitor cells (**NPCs**) as well, both of which are capable of giving rise to oligodendrocytes (**OLs**) *in vivo* and *in vitro* [8]. During the course of MS and murine demyelination models, SVZ cells have shown to proliferate and specify to the oligodendroglial lineage and migrate to lesions, where they actively participate in the endogenous remyelination process [9]–[13]. Besides the fact that the local environment strongly influences NSC differentiation programs [14], molecular mechanisms controlling oligodendroglial differentiation from these undifferentiated cells are not entirely well known.

Transferrin (**Tf**) is an iron transporting protein synthesized in mammalian liver and brain cells [15]–[17]. Together with the choroid plexus [18], OLs are the main Tf synthesizing cells in the brain [19]. Tf is essential for cell survival and it is also known to act as an OL pro-differentiating factor during central nervous system (**CNS**) development [20]–[22] as well as a trophic factor [23], [24]. Transgenic mice over-expressing the human Tf gene [25] showed an increase in their myelin components similar to that found in rats intracranially injected with apoTransferrin (aTf). The *in vivo* effects of aTf were also reproduced in OL primary cultures [26] as well as in N19 and N20.1 oligodendroglial cell lines [27]. Furthermore, aTf can accelerate remyelination in a cuprizone-induced demyelination model [28] and is also able to correct the hypomyelination found in an iron deficient rat model [29], [30].

Although the first remyelination mechanism activated by aTf seems to be oligodendroglial maturation of local oligodendrocyte precursor cells (**OPCs**), no reports have been published describing the effects of aTf on multipotent NSCs/NPCs and their commitment towards the oligodendroglial lineage. Since MS onset is detected in young-adult human beings, in the present work we used young-adult SVZ-derived cells cultured under the form of neurospheres (**NS**) to evaluate Tf's effects in different culture conditions by cell fate analysis.

Our results suggest that aTf participates in the control of oligodendroglial differentiation by two converging regulatory mechanisms: i) in the presence of mitogens, aTf promotes OL lineage commitment from undifferentiated cultures, and ii) after mitogens withdrawal, aTf promotes OL terminal maturation.

## Materials and Methods

### Ethics Statement

The experiments of the current proyect have been made in accordance with national and international recommendations for the care and use of laboratory animals. Approval for these experiments was obtained from the *Ethical Committee For the Care and Use of Laboratory Animals* of the Buenos Aires University School of Pharmacy and Biochemistry. Approval N° 300911-1.

### Animals

Thirty five day old young adult male and female Wistar rats (*Rattus norvegicus*) were housed under 12 hour light/12 hour dark cycle. They were fed rodent pellet chow and water *ad libitum*.

### Culture media

Dulbecco's Modified Eagle Medium: Nutrient Mixture F-12 (DMEM/F12) culture media was prepared by adding 1.2 g of Sodium Bicarbonate and 4 g of anhydrous Glucose per litre. Penicillin G and Streptomycin Sulphate were added to a final concentration of 20 U/ml and 20 µg/ml, respectively, and pH adjusted to 7.4. B27 supplement was added at a 2% final concentration (DMEM/F12-B27). B27 supplement used to prepare the culture media contains unknown-to-the-buyer Tf concentrations. *Proliferation* media was prepared by supplementing DMEM/F12-B27 media with mitogens bFGF (20 ng/ml) and EGF (20 ng/ml). DMEM/F12-B27 media lacking mitogens was considered a *differentiation* media.

### SVZ-derived explant and free-floating neurosphere cultures

Animals were sacrificed and brains were removed. The choroid plexus was removed before SVZ tissue isolation. For SVZ explant cultures, the SVZ tissue was minced to small 1 mm^3^ size fragments by mechanical trituration with a sterile surgical scalpel. Explants were cultured on polyornithine (PO)-coated coverslips for cell adhesion, and maintained for 10 days in DMEM/F12-B27 medium. For SVZ- derived NS cultures, the tissue was mechanically dissociated to a single cell suspension and expanded in *proliferating* media. Growth factors were added to the medium every other day. Proliferating cells grew and aggregated into free-floating NS, which began appearing as from 6 days in culture at 37°C under 5% CO_2_. To amplify NS-derived cells, NS cultures were spun down at 400 rpm for 10 min at room temperature (rt), mechanically dissociated to a single cell suspension and resuspended in fresh proliferating media. Every NS culture was amplified at least twice before plating on an adherent substrate.

### NS fate analysis

Neurosphere cells were attached either without prior dissociation or after NS dissociation. In both situations, 100 µl volume cell samples were plated on PO-coated coverslips within 24-well plastic plates. NS or single cells were incubated at least 6 hs at 37°C – 5% CO_2_ to allow their attachment to the coated coverslip. Once attached, the 24-well plates were completed with 400 µl of fresh *proliferating* media. Cultures were incubated 6 days under *proliferating* conditions in the presence of mitogens (from day 0 to day 6). To determine the cell potential to specific neural cell types, *proliferating* media was replaced by *differentiating* media without mitogens and cultured for 6 additional days (from day 7 to day 12). As from the time of plating (day 0), media was replaced every second day until fixation (day 12). Whole cell protein extracts and culture media samples were used for Western blot analysis. All antibodies used for the immunodetection of various antigens by immunocytochemistry, Western Blot and Cell ELISA are listed in Table 1.

**Table 1:** Antibody specificity.

Specificity	Species	Clonality	Source
BrdU (5-Bromo-2′-deoxyuridine)	Mouse	Monoclonal	Roche
GFAP (Glial Fibrilary Acidic Protein)	Chicken	Polyclonal	Neuromics
Nestin	Chicken	Polyclonal	Neuromics
NG2 (Chondroitin Sulphate Proteoglycan)	Rabbit	Polyclonal	Millipore
PDGFRα (Platlet derived Growth Factor Receptor α)	Goat	Monoclonal	Neuromics
PDGFRα (Platlet derived Growth Factor Receptor α)	Rabbit	Polyclonal	Santa Cruz
MBP (Myelin Basic Protein)	Rabbit	Polyclonal	†
Tf (Transferrin)	Rabbit	Polyclonal	††
CD71 (Transferrin Receptor)	Mouse	Monoclonal	BD Parmingen
BLBP (Brain Lipid Binding Protein)	Rabbit	Polyclonal	Milliopore

† Anti rat MBP polyclonal antibody was a kind gift from the Campagnoni Laboratory (UCLA, California, LA, USA).

†† Anti rat Tf polyclonal antibody was a king gift from the Zakin Laboratory (Institut Pasteur, Paris, France).

### Apo-transferrin treatments

Culture treatments with aTf were done in either *proliferating* or *differentiating* conditions. An aTf sterile 50× stock solution (5 mg/ml) was used to treat cultures at a 100 µg/ml final concentration. Media was replaced every other day during treatment. All the aTf treatments were performed in parallel with control cultures lacking aTf. *Proliferating* media was used during the first 6 days after plating (from day 0 to day 6), and *differentiating* media was used during the following 6 days (from day 7 to day 12).

Nomenclature indicates whether Control (CTL) or aTf treatment (Tf) was used, and the suffix P (for *proliferation*) or D (for *differentiation*) indicate when treatments were installed. CTL_P_CTL_D_ refers to cells cultured from day 1 to 6 with Control *proliferating* media, and from day 7 to 12 with Control *differentiating* media. Tf_P_CTL_D_ condition belonged to cells treated with aTf-containing *proliferating* media from days 1 to 6, but differentiated in a Control condition from day 7 to 12. The CTL_P_Tf_D_ abbreviation indicates cultures grown from day 1 to 6 in Control *proliferating* media, but differentiated from day 7 to 12 in the presence of aTf. The CTL_P_ condition indicates when cells were cultured from day 1 to 6 in Control *proliferating* media and Tf_P_ is used to indicate cells grown in *proliferating* media in the presence of aTf. Neither CTL_P_ nor Tf_P_ cultures are allowed to differentiate. The CTL_D_ condition indicates cells cultured from day 1 to 6 in Control *differentiation* media and Tf_D_ cells cultured from day 1 to 6 in aTf-treated *differentiating* media. For Tf uptake studies, attached cells were exposed for 24 hs prior to fixation with Texas Red-labelled Transferrin (Invitrogen) at a 100 µg/ml final concentration in the culture media.

### MTT assay for cell viability evaluation

Cells were seeded in 96-well plates at a density of 1.10^4^ cells per well in the presence or absence of aTf for 6 days. Cultures were incubated with 10 µl of 5 mg/ml Thiazolyl Blue Tetrazolium Bromide (MTT) substrate for 3 hs at 37°C–5% CO_2_ prior to treatment termination. MTT cleavage to insoluble Formazan crystals (mainly by mitochondrial dehydrogenases) is an indirect measure of cell metabolic activity. The assay was finalized by adding 100 µl of a 10% SDS, 1 N HCl solution. Absorbance was measured with an Amersham Biotrack™ II Visible Plate Reader at 570 nm wave length.

### Cell death analysis by Propidium Iodide staining

Adherent cell cultures were grown on glass coverslips, treated under different conditions and exposed to a Propidium Iodide (PI) solution (100 µg/ml) for 30 min before fixation. Cells were rinsed with 0.1 M Phosphate Buffered Saline (PBS, 8 g/L NaCl, 0.2 g/L KCl, 0.24 g/L KH_2_PO_4_, 1.44 g/L Na_2_HPO_4_, pH 7.4), and fixed with 4% Paraformaldehyde (PFA) – PBS solution for 20 min at rt. Cells were rinsed and mounted with Mowiol® 4–88 anti-fade mounting solution on to glass slides. Cells incorporating the fluorescent PI dye were considered dead cells.

### Cell ELISA for BrdU incorporation quantitation

To study the cell proliferation status, NS cultures were centrifuged at 400 rpm for 10 min. Pellets were resuspended in DMEM/F12-B27 and mechanically dissociated in either *proliferating* or *differentiating* media. Cells were placed in 96-well plates at a density of 1.10^4^ cells per well in the presence or absence of aTf. Cultures were maintained for 6 days, and media was replaced every second day. During the last 24 hs before fixing, cells were incubated in the presence of 0.1 mM 5-Bromo-2′-deoxyuridine (BrdU). After that, medium was discarded and attached cells were washed twice with pre-warmed PBS, fixed in 70% ethanol for 10 min at rt and processed for BrdU immunodetection. Briefly, after rinsing in distilled water, cells were incubated with 2 N HCl for 10 min at 37°C, neutralized with 0.1 M Sodium Borate (pH 9) for 15 min at 37°C and blocked overnight in a 2% Fetal Calf Serum (FCS), 0.1% Triton X-100 solution at 4°C. The anti-BrdU antibody was diluted in a 0.05% Tween 20, PBS solution and incubated for 1 h at 37°C. Cells were then washed in a 0.05% Tween 20, PBS solution. A Horse Radish Peroxidase (HRP)-conjugated secondary antibody was used for 1 h at 37°C. Cells were rinsed in 0.05% Tween 20, PBS and incubated in the dark with a 2 mg/ml OPD substrate and 1 µl/ml H_2_O_2_ in a 0.1 M Citric Acid, 0.1 M Na_2_HPO_4_ buffer for 30 min at rt. The reaction was stopped by adding 50 µl of a 4 N H_2_SO_4_ solution and its absorbance measured at 450 nm wave length with an Amersham Biotrack™ II Visible Plate Reader.

### Immunocytochemistry

A 4% PFA solution was used for cell fixation of attached cultures or free-floating NS. Fixed cells were blocked and permeabilized in 5% FCS, 1% Triton X-100, PBS for 1 h at rt. Primary antibodies were incubated overnight at 4°C. Coverslips were rinsed in PBS, and secondary fluorophore-conjugated antibodies were incubated for 2 hs at rt. All antibodies were prepared in a 1% FCS, 0.1% Triton X-100, PBS solution. Secondary antibodies were diluted together with Höechst nuclear dye. Finally, coverslips were rinsed in deionized water and mounted on glass slides with Mowiol® 4–88 anti-fade mounting solution. For BrdU incorporation analysis by immunocitochemistry, 10 µM BrdU was added to culture media during the last 24 hs before fixation. Cells were fixed in 4% PFA for 20 min at room temperature. Cells were then rinsed in PBS and exposed to 2 N HCl for 20 min at 37°C, neutralized with 0.1 M Sodium Borate (pH 9) for 15 min at 37°C, and blocked overnight in a 2% Fetal Calf Serum (FCS). After the FCS blocking step, the immunocytochemistry procedure with primary and secondary antibodies continued as described above.

### Western Blotting

For protein analysis, adherent cells were washed twice with pre-warmed 0.1 M PBS at 37°C, scrapped off the Petri dishes and lysed in TOTEX buffer (20 nM Hepes pH 7.9, 350 nM NaCl, 20% Glycerol, 1% Igepal®, 1 nM MgCl_2_, 0.5 nM EGTA, 0.1 nM, 10 µg/ml Leupeptine, 10 µg/ml Pepstatine, 0.5 nM DTT, 0.5 nM PMSF) on ice. Total protein extracts solubilized in TOTEX buffer were thoroughly vortexed every 10 min and briefly sonicated before storage at −20°C. Whenever culture media was replaced on adherent cultures, samples of the old culture media were retrieved for protein analysis. These culture media aliquots were centrifuged for 10 min at 10,000 rpm on a bench table MiniSpin*plus* centrifuge to remove any cell debris, and supernatants were recovered for storage at −20°C. Culture media proteins supernatants and to whole cell lysates were quantitated by the Bradford assay, and were electrophoresed using a Mini PROTEAN® 3 System in denaturating 1.5 mm-thick, 12% Polyacrylamide gels containing SDS. Proteins were then transferred onto a methanol-activated Immobylon™ PVDF membrane. Once transferred, membranes were blocked in a 5% skimmed milk, PBS solution for 2 hs at rt. Membranes were then incubated with primary antibodies over night at 4°C. After rinsing in a 0.1% Tween 20, PBS solution, membranes were incubated for 2 hs at rt with HRP-congugated secondary antibodies, and then rinsed in a 0.1% Tween 20, PBS solution. Primary and secondary antibodies were diluted in a 1% FCS, 0.1% Tween 20, PBS solution. Protein immunodetection was performed with a 0.1% 3,3′-Diaminobenzidine (DAB), 0.1% NiCl_2_, 0.1 M Sodium Acetate buffered solution (pH 5), with freshly added Hydrogen Peroxide (1 µl/ml). After colour development, membranes were rinsed in PBS, air dried and scanned. Immunolabeled bands on membranes were quantitated with ScionImage software. For MBP/GAPDH ratio quantitation, all four immunopositive for MBP were pooled into a single densitometric value before normalizing to GAPDH.

### Neurosphere radius size measurement

To evaluate aTf effects on NS formation, NS primary cultures were mechanically dissociated to single cells. Identical aliquots of the cell suspension were amplified in control *proliferating* medium in the absence (CTL) or in the presence of aTf (Tf). Free-floating NS growing in flasks were photographed by adapting a Sony® DSC-WX1 Cybershot camera to an Olympus Tokyo inverted microscope. The radius of at least 200 NS per condition was measured from digital images at different time points with Image Pro® Plus 5.1 software.

### Microscopy image quantitation and statistical analysis

MBP^+^, BrdU^+^ and NG2^+^ cell numbers were counted on 200 and 400 magnification images and relativized to the total Höechst^+^ nuclei. MBP expression semi quantitation in whole 200 magnification images or on individual OLs, in 400 magnification images, was performed by measuring fluorescence Integrated Optical Density (IOD) or fluorescence area with Image-Pro® Plus Software.

Western Blot, ELISA or microscopy image data was analyzed with the GraphPad Prism® using either two-tailed unpaired Student's t Test with a 95% Confidence Interval, One Way ANOVA or Two Way ANOVA.

## Results

### SVZ-derived neurosphere culture characterization

**Figure 1:**
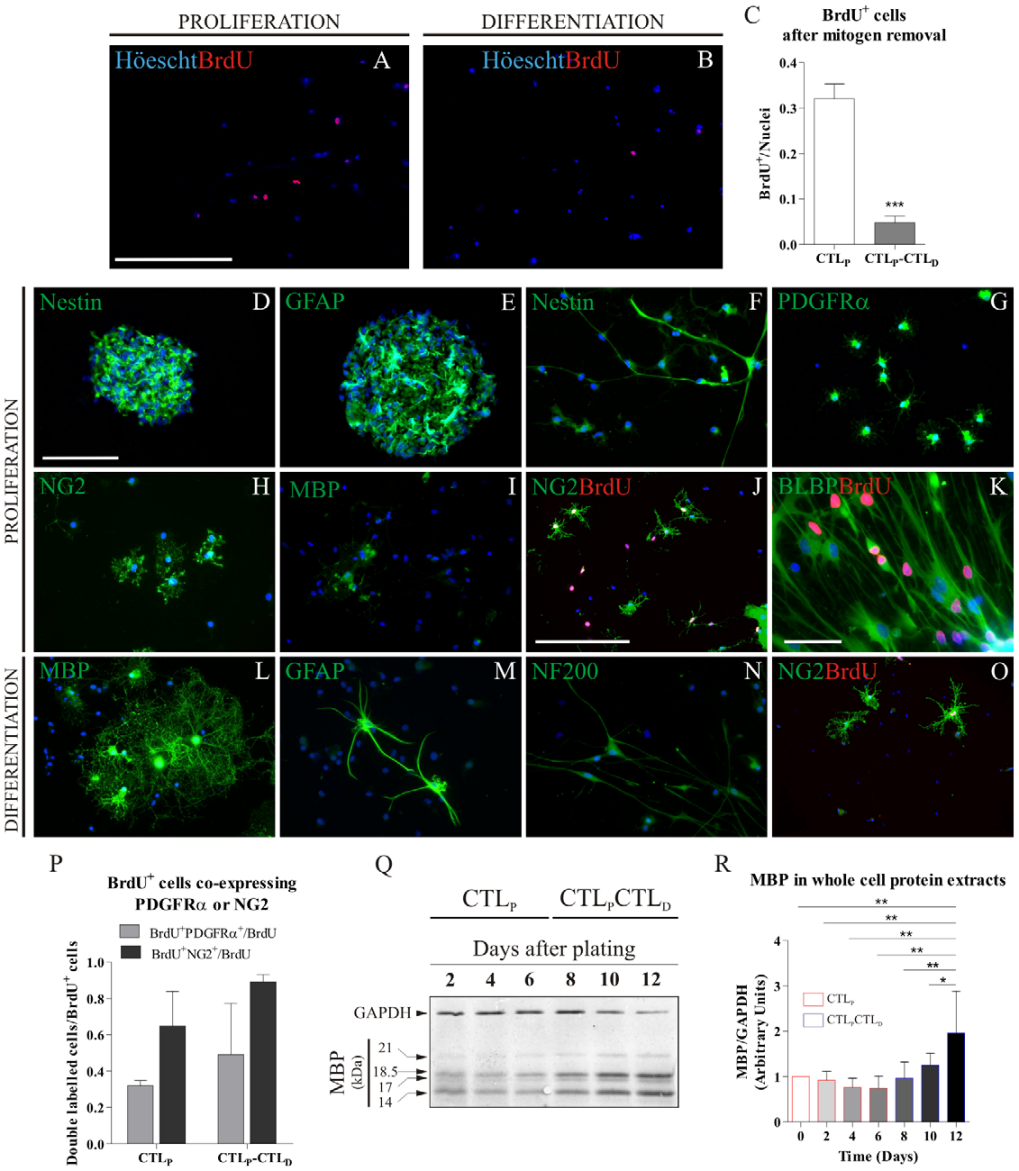
Neurosphere (NS) culture characterization. Proliferation rates under different conditions are shown in *A*–*C*. BrdU incorporation (red) during *proliferation* (CTL_P_, A) or *differentiation* (CTL_P_CTL_D_, B). BrdU^+^ cells are expressed as a percentage of total nuclei for either condition in *C*. Free floating NS during *proliferation* express Nestin (D, green) and GFAP (E, green). After dissociation, NS-derived cells continue to express Nestin (F, green). PDGFRα^+^ (G, green) and NG2^+^ cells (H, green). Few MBP^+^ (I, green) cells were found under *proliferative* conditions. A large proportion of BrdU incorporating cells (J, red) co-expressed with NG2 (J, green). Some BLBP^+^ cells (K, green) incorporated BrdU (K, red). After *differentiation* (L–O), MBP^+^ cells were found with a highly branched and complex morphology (L, green). Cells expressed GFAP (M, green), as well as the neuronal NF200 marker (N, green). BrdU incorporating (O, red) cells were mostly NG2^+^ (O, green) during *differentiation* conditions. BrdU^+^ cells co-expressing NG2, as a proportion of total BrdU^+^ cells, are shown in *P* for either culture condition. A representative Western Blot membrane in *Q* shows how MBP levels increase in whole cell protein extracts as cells differentiate. The densitometric analysis of the MBP isoforms/GAPDH ratio of 5 independent experiments was semi-quantitated in *R*. All 4 MBP isoforms were pooled and considered as a single value before normalizing to GAPDH values. Blue colour in images indicates Höechst nuclear dye. Scale bar in A represents 250 µm for A and B. Scale bar in D equals 100 µm in D–I and L–N, scale bar in J equals 250 µm in J and O, and scale bar in K represents 50 µm. Bars in P represent mean values of 2 independent experiments. Bars in C and R represent Mean + SD of 4 and 5 individual cultures, respectively. Student's t Test was used to analyze data in C, while a One Way ANOVA with an SNK Post-test was used to analyze data in R. * p<0.05, ** p<0.01, *** p<0.001.

We established a neurosphere (NS) culture model to study undifferentiated NSC progression to mature oligodendrocytes. We first characterized the mitogenic effect of bFGF (basic Fibroblast Growth Factor) and EGF (Epidermal Growth Factor) on SVZ-derived dissociated NS cells by evaluating BrdU (5-Bromo-2′-deoxyuridine) incorporation. Cell proliferation was stimulated by the addition of mitogens to the culture medium and compared to cultures lacking mitogens. After culturing attached cells in control *proliferation* media (CTL_P_) for six days, 30% of the total nuclei incorporated BrdU during the last 24 hs before fixation (Fig. 1A,C). Identical cultures show more than 80% of the total nuclei were BrdU^+^ after exposition to BrdU for 6 days under proliferating conditions (not shown). After cells were switched to a culture media lacking growth factors for an aditional 6 days (CTL_P_CTL_D_), BrdU^+^ cells decreased to 5% of total nuclei (Fig. 1B, C). We further evaluated the expression of different cell markers in this culture system. Floating NS expressed the Neuroepithelial Stem Cell Protein, Nestin (Fig. 1D), and Glial Fibrilary Acidic Protein (GFAP) (Fig. 1E), characteristic of type B undifferentiated NSCs. After NS dissociation, plating and culturing under *proliferation* condition, most cells expressed Nestin (Fig. 1F), the OPC marker Platelet Derived Growth Factor Receptor α (PDGFRα) (Fig. 1G) and/or the polydendrocyte marker Chondroitin Sulphate Proteoglycan 4, NG2 (Fig. 1H). A small proportion of cells expressed the mature OL marker Myelin Basic Protein (MBP) (less than 3%) (Fig. 1I). Colocalization analysis revealed that in these proliferating cultures, more than 50% of BrdU^+^ cycling cells were NG2^+^ (Fig. 1 J, P). A few proliferating cells also expressed the radial glia marker Brain Lipid Binding Protein (BLBP) (Fig. 1K). After 6 days under *proliferation* and 6 additional days under *differentiation*, we observed an increase in the number of MBP expressing cells (at least 10%) (Fig. 1L). Furthermore, we detected GFAP^+^ astrocytes (Fig. 1M) and Neurofilament heavy chain positive (NF200^+^) neurons (Fig. 1N), demonstrating that cells in SVZ-derived cultures are able to commit to different neural lineages. Cells expressing NG2 were detected under *differentiation* conditions (Fig. 1O). Almost 80% of BrdU^+^ cells coexpressed NG2 under *differentiation* (Fig. 1O, P), suggesting proliferation of this particular cell type did not depend on bFGF nor EGF addition to the media. Although to a lesser extent than NG2^+^ cells, PDGFRα^+^ cells represented a significant proportion of BrdU+ cells in the CTL_P_CTL_D_ condition as well (Fig. 1P). MBP expression was also evaluated by Western blot analysis in whole cell protein extracts at different time points in CTL_P_CTL_D_ cultures (Fig. 1Q). Densitometric analysis of immuno-positive bands confirmed lower cellular levels of MBP during the *proliferation* condition (Fig. 1R, 0 to 6 days) that correlates with the immunocytochemical data, and a progressive up-regulation of the MBP content during the *differentiation* stage (Fig. 1R, days 8 to 12). At the end of *differentiation* we detected a two fold increase in MBP expression levels compared to those found in the initial culture.

We also cultured intact NS (without dissociation) under the same *proliferation* and *differentiation* conditions. During proliferation, 75–85% of cells incorporated BrdU (Supp Fig S1A). Cells derived from attached NS migrated away from high nuclear density regions and spread out evenly in a radial manner (Supp Fig S1B). Together with the appearance of Nestin^+^ and GFAP^+^ cell processes which stretched out from the centre of the NS (Supp Fig S1C,D), numerous cells initiated their migration in a radial manner away from the NS. A few Nestin^+^ cells incorporated BrdU (Supp Fig S1C, whole arrow heads) while others did not (Supp Fig S1C, empty arrow heads). Some cells expressed MBP and were located at a distance from the NS center (Supp Fig S1D). After incubation in *differentiation* media we observed a reduction in the percentage of BrdU^+^ cells (Supp Fig S1E). GFAP^+^ astrocyte cell bodies were located within the attached NS, with processes stretching out of the cellular mass (Supp Fig S1F). Mature OLs arise at a distance from the NS surface (Supp Fig S1G). NG2^+^ progenitors were found closer to the NS surface than MBP^+^ cells (Supp Fig S1H). GFAP expressing cells were also observed at a distance from the centre of the NS together with mature OLs (Supp Fig S1I). Cell differentiation towards the oligodendroglial lineage seems to occur following a specific spatial distribution in regards to the attached sphere.

### Distinct effects of aTf in SVZ-derived proliferating and differentiating cultures

**Figure 2:**
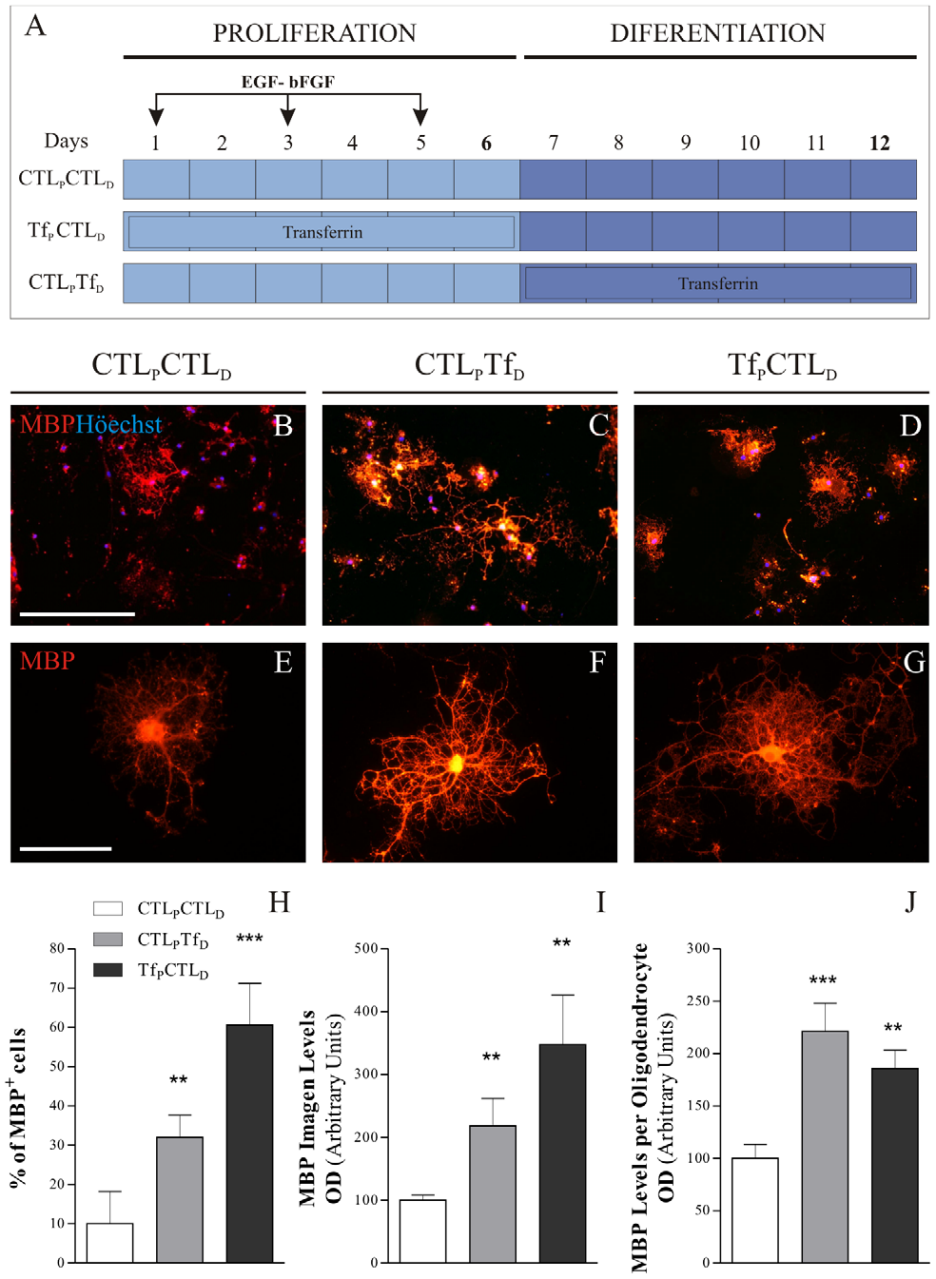
ApoTransferrin treatment promotes oligodendroglial commitment and maturation. A schematic representation of the strategy used for culture treatments is shown in A. The OL marker MBP indicates mature oligodendrocytes (red). Low magnification images of MBP^+^ cells are shown in B, C and D. Images E, F and G show representative high magnification images of MBP^+^ cells. MBP^+^ cell numbers in *H* are a percentage of total nuclei. Whole image MBP fluorescence optical density (OD) was quantitated and represented in I. Individual MBP^+^ cell images were used to semi-quantitate the protein expression levels per OL as indicated in J. ApoTransferrin treatment increases both MBP^+^ cell numbers and MBP protein levels (I,J). Blue colour in images indicates Höechst nuclear dye. Scale bar in B represents 250 µm for B, C and D, and scale bar in E represents 50 µm in E, F and G. Bars in all graphs represent Mean + SD of 2 independent experiments. ** p<0.01, *** p<0.001.

We next tested the functional effects of exogenous aTf on NS cell cultures. ApoTf treatment was performed as schematized in figure 2A, using a standard differentiation protocol in which dissociated NS cells were cultured for 6 days under the *proliferation* condition, followed by 6 days in *differentiation* medium. Oligodendrocyte maturation was evaluated by MBP immunocytochemical detection. In CTL_P_CTL_D_ cultures, 10% of cells were MBP^+^ (Fig. 2B) and increased to 30% in the CTL_P_TF_D_ condition (Fig. 2C,H), and indicates aTf acts on OL generation/maturation during *differentiation* condition.

In addition to aTf effects during cell *differentiation*, we found that mature OL proportions also varied substantially after aTf treatment during the *proliferation* condition followed by *differentiation* in the absence of aTf (Tf_P_CTL_D_), since we observed a 6 fold increase in MBP^+^ cell numbers relative to control cultures (CTL_P_CTL_D_) (Fig. 2D,H). No significant changes were found in MBP^+^ cells numbers at the end of the *proliferation* condition amongst control and aTf-treated cultures (CTL_P_ vs. Tf_P_), indicating that new mature OL found at the end of the assay are exclusively generated during the *differentiation* period. The fact that aTf enhanced OL generation when added to the media during *proliferation* suggested that aTf could be exerting an additional effect on undifferentiated NSC/NPC, glial restricted progenitors (GRPs) or OPCs, the latter cell types having high proliferation rates under *proliferating* cultures. Moreover, MBP expression levels were analyzed by whole image fluorescence semiquantitation and showed an upregulation in CTL_P_Tf_D_ and Tf_P_CTL_D_ cultures compared to CTL_P_CTL_D_ (Fig. 2I). Higher magnification images of individual OLs were analyzed in terms of their MBP expression levels and morphological complexity. This analytical approach demonstrated that both CTL_P_Tf_D_ and Tf_P_CTL_D_ OLs expressed higher MBP levels and exhibited a far more complex morphology compared to CTL_P_CTL_D_ OLs (Fig. 2E–G,J). There were no changes in the proportions of MBP^+^ cells between control and aTf-treated cultures at the end of proliferation stage (data not shown), suggesting these newly formed OLs were exclusively generated during the *differentiation* condition.

Similar findings were found after aTf treatment of intact NS (Supp Fig S2A). Semi-quantitative analysis of images belonging to NS derived cells revealed a significant increase in MBP levels after aTf treatment both during *differentiating* (CTL_P_Tf_D_,Supp Fig S2B, D, E) and *proliferating* (Tf_P_CTL_D_, Supp Fig S2C, G, F) conditions. Cells derived from intact NS are equally responsive to aTf and could serve as an alternate culture system for these kinds of studies.

### ApoTf effects on cell death and proliferation

**Figure 3:**
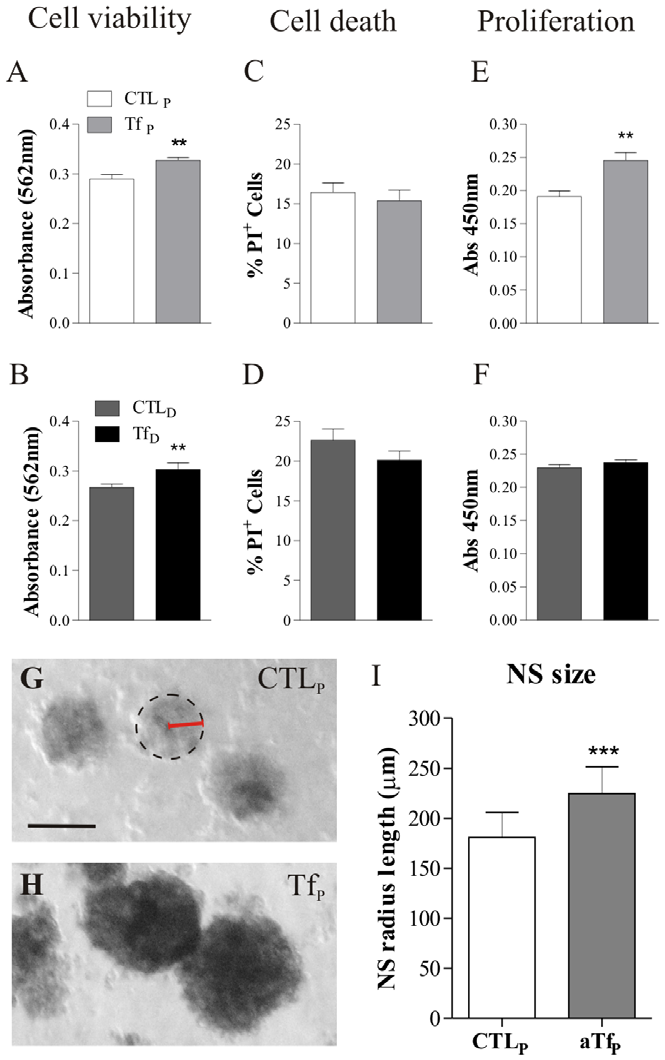
Changes in cell viability, death and proliferation after aTf exposure. Cell viability, death and proliferation during *proliferating* conditions are shown in A, C and E, respectively. Cell viability, death and proliferation during *differentiation* conditions are shown in B, D and F, respectively. Cell viability was evaluated by MTT, cell death was evaluated by Propidium Iodide (PI) incorporation, and proliferation was evaluated by Whole Cell ELISA. The radius of free floating NS was measured on 2D images of NS as shown in *G*. Dotted circle over-laid on a NS image in *G* indicated the NS border, and NS radius is labelled in red. After a 9 days in culture, the average size of NS was larger in aTf-treated cultures compared to controls (G and H). The quantitation of NS radius length in each condition is shown in *I*. Scale bar in G represents 100 µm in G and H. Bars in A to F represent Mean + SEM, while data in I represents Mean + SD of 4 independent experiments. Student's t Test was used to analyze data in *A–F* and a Two Way ANOVA for the data reprersented in *I*. ** p<0.01, *** p<0.001.

To further investigate the mechanisms by which aTf could cause a net increase in mature OL numbers, cell viability, cell death and BrdU incorporation were evaluated during cell *proliferation* or *differentiation*. Cell viability was evaluated by MTT assay. ApoTf had a significant effect on the metabolic cell activity, either when added during *proliferation* or *differentiation* stages. Both aTf-treated cultures (Tf_P_ and Tf_D_) had higher formazan production levels compared to their controls (CTL_P_ and CTL_D_, respectively) (Fig. 3A, B). Since the increase in metabolic activity could be a consequence of higher cell viability or an increase in cell proliferation, we analyzed these two processes after aTf treatment. Cell death was evaluated with Propidium Iodide (PI) fluorescent probe. The PI assay revealed no significant changes in the percentage of PI^+^ cells amongst proliferating cultures (CTL_P_ and Tf_P_, Fig. 3C), nor between differentiating cultures (CTL_D_ and Tf_D_, Fig. 3D). The overall PI^+^ cell numbers decreased after mitogen removal from culture media regardless of the aTf supplementation, resembling the normal selection process that takes place *in vivo* during normal tissue development (Fig. 3C and D). BrdU incorporation was evaluated by whole cell ELISA to determine whether aTf affected cell proliferation. When aTf was added to the culture media during *proliferation* (Tf_P_), cultures exhibited a significant increase in overall BrdU incorporation compared to cultures lacking aTf (CTL_P_) (Fig. 3E). On the contrary, when aTf was present during *differentiation*, no significant changes between CTL_D_ and Tf_D_ were observed (Fig. 3F). These results suggest a dual role of aTf in the culture progression regulation. While acting as a pro-maturating factor on OLs undergoing differentiation, Tf seems to promote cell division in proliferating cultures. We further evaluated aTf effects on the formation and size of floating NS, and found aTf addition to NS during *proliferating* media resulted in an increase in the mean radius length of NS (Fig. 3G–I). The mean radii length difference between Tf-treated and non- treated cultures increased with time between 5 and 7 days in culture (Supp Fig S3). We consider aTf is able to enhance the proliferation rate of NS-forming cells in the presence of mitogens, and this increase in cellularity is responsible for the increase in the NS size.

### Identification of aTf cell targets

**Figure 4:**
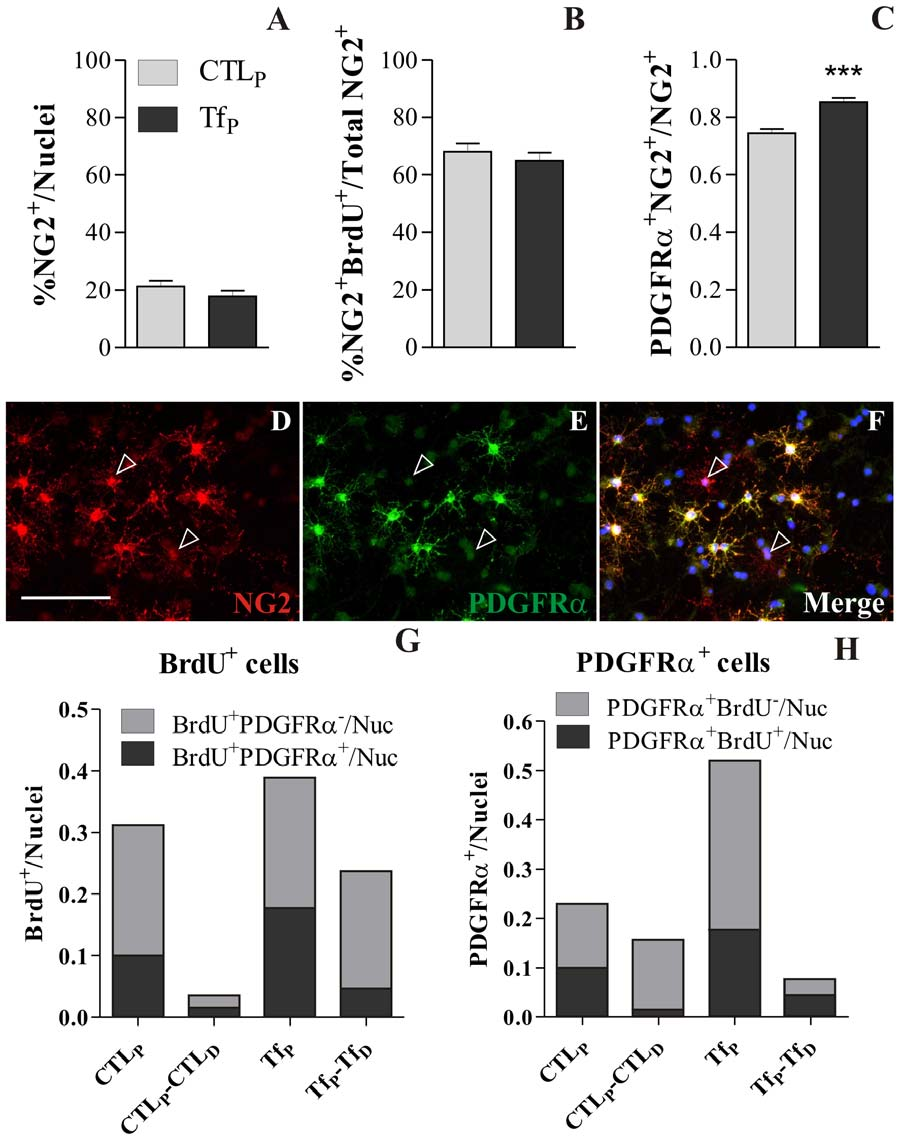
aTf effects on NG2^+^ and PDGFRα^+^ cell populations during *proliferation*. NG2^+^ cell numbers are expressed as a percentage of total nuclei (A). BrdU^+^ cells co-expressing NG2 are represented as a percentage of total NG2^+^ cells (B). A significant increase was observed in NG2^+^ cells that began co-expressing PDGFRα after aTf exposure during *proliferation* (C). NG2 expression (D, red) was found in process-bearing cells with a relatively complex morphology. PDGFRα expression (E, green) was seen in cells with the same morphology as NG2^+^ cells. NG2 and PDGFRα are merge with Höechst in *F*. Empty arrowheads indicate NG2^+^ cells lacking PDGFRα expression. The proportions of BrdU^+^ and PDGFRα^+^ cells under different culture contitions are shown in G and H, respectively. Scale bars in D represents 100 µm for D–F. Bars in A–C represent Mean + SEM, while bars in G and H represent Mean values of two independent experiments. *** p<0.001.

In order to identify potential cellular targets of aTf in *proliferating* cultures, we first evaluated NG2 expression as a marker of bipotential polydendrocytes. There was no significant change in NG2^+^ cell numbers when cultures were treated with aTf during *proliferation* (Tf_P_), indicating that aTf did not seem to act through the amplification of the NG2^+^ cells pool (Fig. 4A). NG2^+^ are rapidly expanding progenitors in vitro, so we counted NG2^+^ cells that co-labelled with the anti BrdU antibody within the NG2 population. Tf_P_-treated cultures showed no significant changes in the number of NG2^+^/BrdU^+^ cells compared to CTL_P_ (Fig. 4B). Nonetheless, whether cells expressing NG2 maintain a constant BrdU^+^ proportion in the presence of aTf does not say much about their potentiality nor their possible commitment to the oligodendroglial lineage after aTf treatment. Further analysis of the OPC marker PDGFRα^+^ showed that these cells were a subpopulation within the NG2^+^ cell pool in our culture system. Colocalization analysis after aTf treatment showed an increased proportion of PDGFRα^+^/NG2^+^ double labelled cells at the expense of a reduction in the numbers of PDGFRα^−^/NG2^+^ cells (Fig. 4C, D). These results suggest that NG2^+^ polydendrocytes are narrowing their potential towards an OL restricted phenotype by activating PDGFRα expression in response to Tf. Representative images of proliferating cells in the control condition expressing NG2 and PDGFRα show most cells co-expressing both cell surface markers, although a fraction of cells are NG2^+^/PDGFRα^−^ (Fig. 4D–F).

The increased proliferation rate of the SVZ-derived cells *in vitro* by aTf was confirmed by immunocytochemistry (Fig. 4G) during *proliferation* (Fig. 4G, CTL_P_ and Tf_P_). Furthermore, characterization of BrdU^+^ cells revealed that aTf treatment increased the proliferation of PDGFRα^+^ cells, sionce we detected more BrdU^+^/PDGFRα^+^ double labelled cells in Tf_P_ compared to CTL_P_ (Fig. 4G). Contrarily, Tf treatment under the *differentiation* condition seems to decrease the proportion of proliferating cells that express PDGFRα, although the overall proliferation is increased compared to controls (Fig. 4G, CTL_P_CTL_D_ and Tf_P_Tf_D_).

We further analyzed the PDGFRα^+^ population in order to determine how the proliferation of this particular cell type is affected by aTf treatment. When evaluating the total number of PDGFRα^+^ cells, they were increased in Tf_P_ compared to CTL_P_, and both PDGFRα^+^/BrdU^+^ and PDGFRα^+^/BrdU^−^ cells proportions were equally incremented (Fig. 4H). During *differentiation*, aTf decreases the number of PDGFRα^+^ cells, although the proportion of proliferating PDGFRα^+^ cells increases respect to controls (Fig. 4H, CTL_P_CTL_D_ and Tf_P_Tf_D_).

### Tf receptor immunoreactivity in SVZ-derived cultures

**Figure 5:**
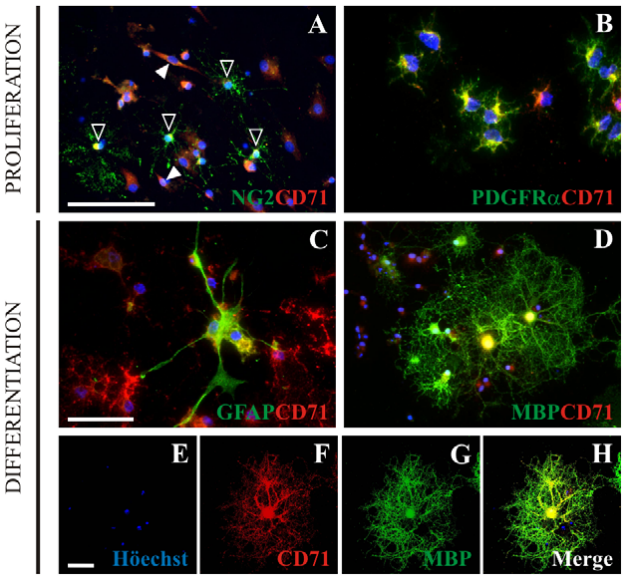
Transferrin Receptor (TfRc) expression. TfRc was immunodetected with the CD71 monoclonal antibody. During *proliferation*, NG2^+^ cells (A, green) and PDGFRα^+^ cells (B, green) co-express the TfRc (red). Empty arrowheads in A indicate NG2^+^/CD71^+^ cells. Filled arrowheads indicate bipolar-shaped NG2^−^/CD71^+^. After *differentiation*, GFAP^+^ (C, green) cells were found expressing TfRc (red). MBP^+^ cells (D, green) show strong CD71 immunolabelling (red). Confocal image analysis reinforces the concept of MBP^+^ OL expressing the TfRc (E–H). The blue colour in images indicates Höechst nuclear dye. Scale bar in A equals 100 µm in A and D, scale bar in C equals 50 µm, and scale bar in E equals 40 µm for E–H.

Since the canonical mechanism of cellular uptake of Tf is mediated by Tf Receptor (TfRc), we evaluated the presence of TfRc in dissociated NS cells at different time points during culture progression by immunocytochemistry with the anti CD71 monoclonal antibody. These descriptive results confirm that the TfRc was detected at low or moderate levels in all cell types *in vitro*. At the end of the *proliferation* condition CD71^+^ cells co-localized with the progenitor marker NG2 (Fig. 5A, empty arrowheads) and the OPC marker PDGFRα (Fig. 5B). TfRc expression in these cells was restricted to a peri-nuclear region in the cell soma. Other CD71^+^ cells were also detected which did not express either NG2 nor PDGFRα, and had a bipolar morphology (whole arrowheads in Fig. 5A). After *differentiation*, GFAP^+^ cells expressed low TfRc levels (Fig. 5C), and intermediate levels were found in MBP^+^ OLs (Fig. 5D). We used confocal images to show TfRc immunostaining colocalized with MBP^+^ cells, and that the former was not only located on the cell body surface, but expressed on process membranes as well (Fig. 5E–H). In order to determine the presence of TfRc in the SVZ tissue, we used SVZ explants cultures to avoid the undesired CD71 high expression normally seen in vasculature *in vivo*. Although tissue explants are grown *in vitro*, most of the cytoarchitecture remains unchanged, rendering the cellular environment somewhat similar to the one found in the original tissue. In these cultures, TfRc immunoreactivity was mainly found in cells within the explants itself (Supp Fig S4A), while Tf^+^ cells were found on cells that had migrated away from the explant (Supp Fig S4B), where a few cells displayed simultaneous Tf and TfRc immunoreactivity (Supp Fig S4C, C′). Thus, Tf signalling is actually present in the cells derived from the lateral ventricle wall, which could effectively be functional Tf cell targets.

### Transferrin uptake

**Figure 6:**
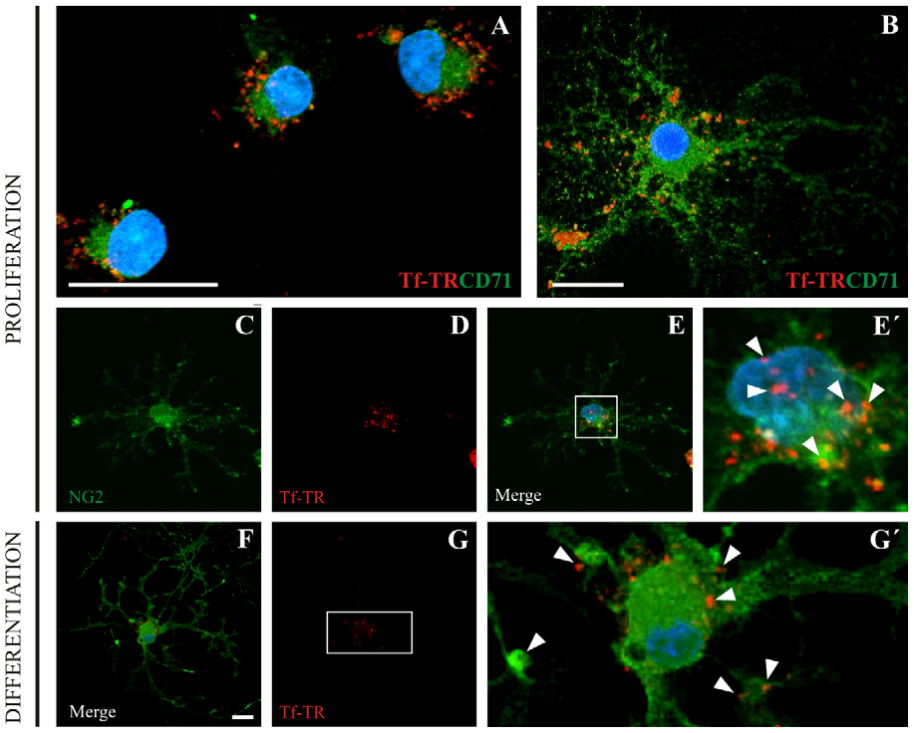
Transferrin – Texas Red fluorescent conjugate (Tf-TR) to follow Tf cellular uptake. Cells expressing the TfRc (A, B, green) show Tf-TR incorporation (red) during *proliferation* in cells with diverse morphology. NG2^+^ cells (C, green) also incorporate Tf-TR (D, red). Images *C* and *D* are merged in *E*, and the inset in *E* is shown at a higher magnification in *E′*. Arrowheads in *E′* indicate the Tf-TR punctate pattern within the cell. Image F shows MBP, Tf-TR and Höechst overlap on OL during differentiation. Tf-TR is shown in G. The inset in G is magnified in G*′*. Arrowheads in *G′* indicate Tf-TR detection in the OL cell body and in one of its ramifications. The blue colour in images indicates Höechst nuclear dye. Scale bars in A and B equal 20 µm, scale bar in C equals 20 µm for C–F and G.

Cultures were treated with a Transferrin-Texas Red fluorescent conjugate (Tf-TR) during *proliferation* or *differentiation* during 24 hr, and its incorporation was evaluated by Texas Red fluorescence detection. We observed a cytoplasmic punctate pattern characteristic of endosomal vesicle trafficking, which was detected in CD71^+^ cells (Fig. 6A, B). Under the *proliferation* condition, all NG2^+^ cells were Tf-TR^+^ (Fig. 6C–E, E′). However, Tf-TR was not evenly distributed amongst these cells; some cells had few fluorescent puncta, while others only had faint flurescent puncta within their cytoplasm. MBP^+^ cells showed different patterns of Tf-TR internalization (Fig. 6F–H, H′), where some MBP^+^ OLs had endosomal vesicles restricted at the end of their processes while others displayed a generalized punctate cytoplasmic pattern.

### Release of endogenous Tf to the culture medium is downregulated during culture differentiation

**Figure 7:**
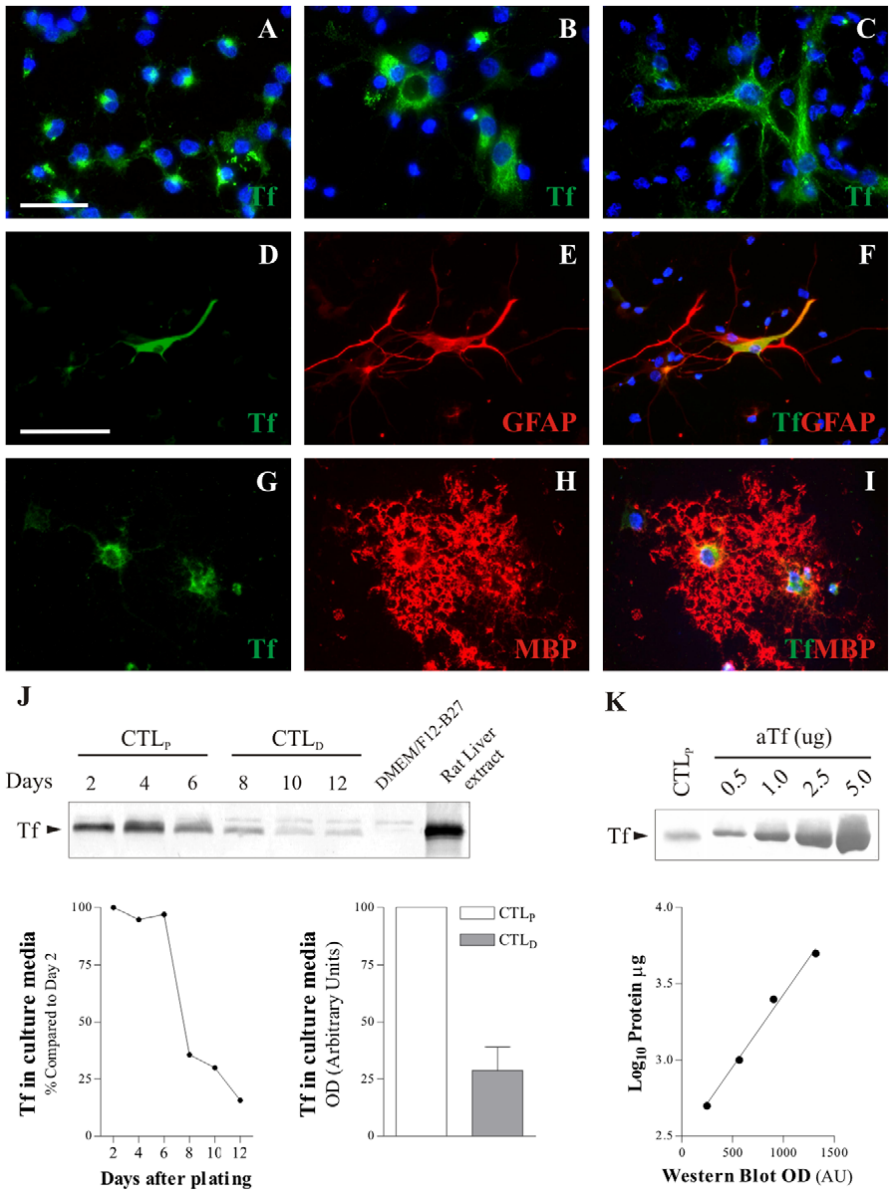
Endogenous Tf synthesis and secretion to culture media. Tf immunodetection (green) was observed in discrete cells with heterogeneous morphologies during *proliferation* (A–C). During *differentiation*, Tf (D, green) was observed in some GFAP^+^ cells (E and F, red). MBP^+^ cells (red) were found to be Tf^+^ (green) as well (G–I). Culture media proteins were electrophoresed, blotted and immunolabelled against Tf (J). Tf was observed in the culture media samples at different time points, and decreased once cells were switched from the *proliferating* to the *differentiation* condition. Western Blot (WB) bands positive for Tf were quantitated and represented in a graph to compare Tf protein levels in culture media at the end of the *proliferation* and the *differentiation* conditions. Results in *K* show Tf semi-quantitation performed by WB. Known amounts of Tf were blotted and used to build a standard curve of different protein masses as a function of the WB band optical density (OD). Tf in culture media samples were interpolated in the standard curve. Data in *J* is a representative image of 3 individual experiments. The blue colour in images indicates Höechst nuclear dye. Scale bar in A corresponds to 50 µm in A–C and G–I, while scale bar in D equals 100 µm in D–F. Gray bar in J indicates Mean + SD.

Immunocytochemical analysis of control *proliferating* cultures showed that Tf^+^ cells exhibit diverse morphologies. Some of them had short and spiky processes, while others displayed round or bipolar morphologies (Fig. 7A–C). The Tf^+^ cells in Figure 7A are highly suggestive of NG2^+^ or PDGFRα^+^ cell morphologies, while cells observed in Figure 7C resemble GFAP^+^ cells. At the end of *differentiation*, a subset of Tf^+^ cells expressed GFAP (Fig. 7D–F) or MBP (Fig. 7G–I). Transferrin protein expression was analyzed during progression of *proliferation* and *differentiation* stages. Western blots of protein extracts revealed no significant changes in the intracellular Tf content during *differentiation* compared to *proliferation* (not shown). Surprisingly, we found that Tf was secreted to the extracellular medium during proliferation stages, and this secretion was progressively down regulated during culture differentiation (Fig. 7J). Quantitation analysis of immunopositive bands showed Tf was secreted to the medium during *proliferation* at lower concentrations than the exogenous aTf added to the culture media for treatment (Fig. 7K). These results indicate the presence of Tf secreting cells in our culture system, and strongly suggest that cells other than mature OLs are responsible for this secretion.

## Discussion

Oligodendrocytes in the adult brain can be generated from two main cellular sources. On the one hand, proliferating progenitor cells dispersed throughout the brain act as local precursors. On the other, SVZ NSCs and multipotent progenitors serve as undifferentiated reservoirs that participate in the maintenance of CNS cell populations throughout adulthood and as well as in response to brain injury [2], [31]. Oligodendroglial differentiation from multipotent adult NSCs follows a stepwise pathway in which morphological changes are accompanied by the sequential down regulation of NSC markers and activation of markers belonging to mature myelinating OLs. In this report, we have characterized the progression of cell differentiation using young adult SVZ-derived NS cultures. Cells growing under *proliferating* condition are enriched in actively dividing cells. However, we cannot know for sure if these BrdU^+^ cells are undifferentiated/uncommitted cells or have become specified progenitors that still retain the capacity to enter the cell cycle. We know cells committed to the oligodendroglial cell lineage are able to proliferate [32] so we cannot rule out the possibility that proliferating adult SVZ NSCs and NPCs are already committed to a certain lineage as in embryonic stages [33], [34]. Moreover, we find that more than half of the BrdU^+^ cells undergoing *proliferation* express the polydendrocyte marker NG2 and could be potential OLs [33], [35]–[40]. We therefore conclude proliferating cells in these conditions range from NSCs, NPCs, GRPs and even pre-OLs, all of which have the capability of entering the cell cycle. We must stress that even in the presence of mitogens we were able to find a hand-full of mature MBP^+^ OLs that could have triggered their differentiation and maturation machinery regardless of the growth factors present in the culture media.

Overall BrdU incorporation decreases after mitogen removal. Although we did not expect to find proliferating cells in the *differentiating* condition, a few proliferating cells continue existing in cultures lacking growth factors. Most of these BrdU^+^ cells express NG2, indicating NG2^+^ progenitors still continue to proliferate in the *differentiating* conditions and could be striving to become mature MBP^+^ OLs. Since *in vivo* analysis of NG2^+^ cells demonstrate they are present throughout the entire white and grey parenchyma of young-adult rat brains as a resident population [41], an alternative explanation for the presence of BrdU^+^/NG2^+^ cells *in vitro* during *differentiation*, is that they are permanently arrested as NG2^+^ cells, and would require further extrinsic cues to trigger the oligodendroglial differentiation and maturation machinery. Our results highlight the concept of a heterogeneous population, where proliferating cells are found amongst fully mature cells that divide in an EGF-bFGF independent manner.

The NS culture system allowed us to observe that more MBP^+^ cells are generated after *differentiation* in the presence of aTf compared to controls. Furthermore, individual OLs express higher MBP levels and show a more mature phenotype in the presence of aTf. Since extensive literature has been published describing Tf as a pro-maturation factor, we could assume that aTf in this scenario is promoting OL maturation. A possible explanation for this finding is that aTf could act by stimulating OPCs, pre-oligodendrocytes and/or immature OLs to become mature MBP^+^ cells faster. Thus, if maturation is accelerated in the presence of aTf, it should be expected that mature OLs grown in the presence of aTf will spread, ruffle and branch-out new processes, taking the lead compared to control OL cells. Surprisingly, cells cultured in the presence of aTf during proliferation followed by a six-day period in control *differentiating* medium, show a six- fold increase in the numbers of MBP^+^ cells when compared to untreated cultures.

Although aTf addition increased cell viability relative to controls either during proliferation or differentiation, the increase in cell metabolism is explained by different regulatory mechanisms depending on the time frame during which cells are exposed to aTf. ApoTf treatment during *proliferation* induces cell division without affecting cell survival. On the other hand, aTf does not alter cell death nor proliferation rate during *differentiation*, but rather increases cellular metabolism and/or viability of mature OLs.

The observation that aTf- treated floating spheres grown in *proliferating* conditions increased their size when compared to controls, confirms aTf possess a mitogenic effect on NS-forming cells. Indeed, it has been shown that primary or secondary NS do not develop in Tf- deprived media, indicating that aTf is an essential factor required for NSC proliferation [42].

Despite the fact that BrdU^+^ cells are a heterogeneous population, we do not know if aTf has an effect on a specific proliferating subpopulation or on all proliferating cells, regardless of their cell commitment or differentiation stage. In fact, if aTf enhances proliferation of uncommitted cells, these would expand more readily than controls, although the proportions of different cell types should remain constant once cell commit and mature to a certain lineage. Nevertheless, when cells are treated with aTf during *proliferation*, and are allowed to differentiate under control conditions, higher numbers of OLs are observed than in control cultures. Previous reports demonstrate that aTf inhibits cell cycle progression and leads to an early initiation of OL maturation in OPCs primary cultures [43]. Interestingly, we find that cell fate choice is influenced by aTf addition when cells are maintained in mitogen containing medium. ApoTf promotes PDGFRα expression within NG2^+^ cell populations, indicating a shift in cell potency towards an oligodendroglial-restricted lineage. Unlike earlier studies considering NG2^+^ and PDGFRα^+^ cells as the same cell pool [38], [39], [44], we have found that not all NG2^+^ cells generated from adult NS cultures are PDGFRα^+^. Our results suggest that aTf is able to increase cell specification towards the OL lineage of NG2^+^/PDGFRα^−^ cells through the activation of PDGFRα expression. This mechanism could explain why more OLs are generated in Tf- treated proliferating cultures.

We report cells in our cultures expressing varying levels of the TfRc. NG2^+^ cells showed a lower but distinct TfRc expression. PDGFRα^+^ OPCs were CD71^+^ as well, and mature OLs were both MBP^+^/CD71^+^. The use of fluorescence-labelled Tf demonstrated that NG2^+^ polydendrocytes and MBP^+^ mature OLs readily incorporate the above-mentioned molecule, and is highly suggestive that aTf acts by binding directly to TfRc on these cells. Since Tf is expressed by OL lineage cells, a plausible hypothesis would be that Tf acts on OLs in an autocrine manner. However, even if NG2^+^ and MBP^+^ cell types express TfRc, we cannot be certain if the effects we observe in treated cultures are a direct consequence of aTf binding to TfRc on these cells, or acts on other CD71^+^ cells within our cultures that are NG2^−^ and MBP^−^_,_ and respond to aTf by secreting intermediary molecules that mediate the effects observed on SVZ-derived cultures. Astrocytes and neurons for example, express TfRc and secrete PDGF [45]. A similar model was proposed for OLs in the developing rat optic nerve by Barres and Raff (1994) [46].

Further evaluation of control culture supernatants showed Tf was actively being secreted out of the cells when growing in the presence of mitogens, and decreased a 75% after culturing during *differentiation*. This demonstrates there are cells within SVZ-derived cultures capable of synthesizing Tf, and suggests this Tf could have similar effects as the exogenously added aTf. Even when Tf is being produced in these cultures *in situ*, reproducible and consistent effects can be clearly seen when aTf is added exogenously to the cultures. Once secreted to the extracellular medium, Tf could be mediating autocrine and/or paracrine signalling. We do not disregard endogenous Tf production, but semi-quantitative WB analysis showed that the endogenously produced Tf is lower than the exogenous aTf added to the media. An interesting point of debate rests on the possibility of EGF and/or bFGF modulating Tf secretion form these cells. Furthermore, the fact that Tf is secreted to culture supernatants during proliferation highlights the possibility that an alternate neural cell type can produce Tf *in vitro*, other than mature non-proliferating OLs.

**Figure 8:**
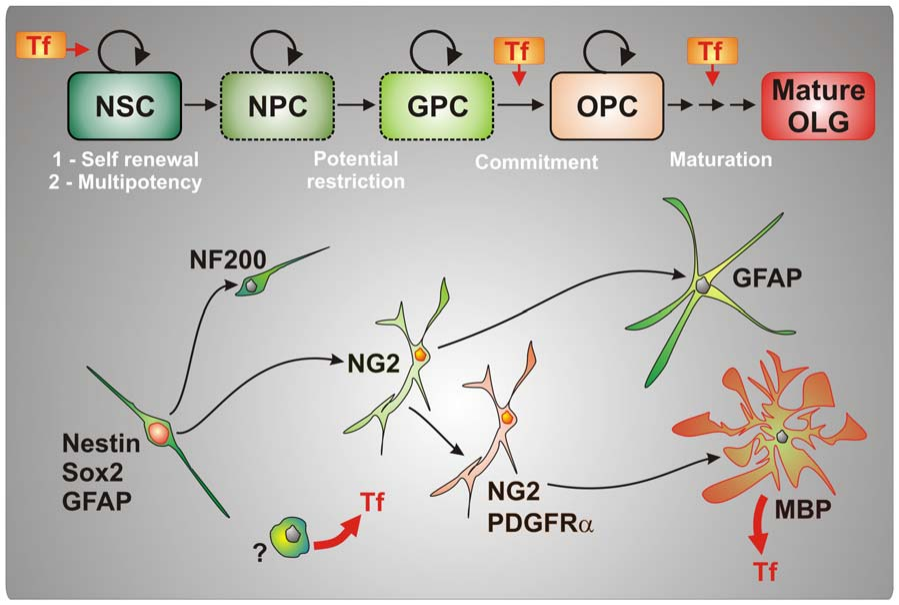
Role of Transferrin during oligodendrogenesis. A hypothetical model of Tf participation during different steps of neural stem cell progression towards mature oligodendrocytes *in vitro*. Tf promotes cell proliferation of neurospheres forming cells. Our results suggest this activation of cell division might occur on undifferentiated neural stem cells (NSC). Tf was also shown to influence the transition of more restricted NG2^+^ neural progenitor cells (NPC) into NG2^+^/PDGFRa^+^ oligodendroglial precursor cells (OPC). Late during oligodendroglial maturation Tf also promotes oligodendrocyte morphological development. In spite of OLs being known to synthesize Tf, the results suggest there are other cells in this culture system, distinct from OLs, which secrete Tf to the culture media.

Taken together, our results reveal that aTf effects on NS cells differ according to their proliferative state *in vitro* as schematized in figure 8. ApoTf participates in the control of a) cell proliferation, b) NG2^+^ polydendrocyte specification towards OPCs and c) OL maturation, in NS cell culture. Considering aTf promotes NS growth without affecting proliferation rates of polydendrocytes or OPC, it is tempting to speculate that, at this early stage, aTf targets might be NPCs, or even NSCs. Under proliferating conditions we also find that aTf participates in the control of undifferentiated cells towards the OL lineage, as indicated by the presence of more double labelled NG2^+^/PDGFRα^+^ cells after aTf treatment. Once cell differentiation is triggered by mitogen withdrawal, aTf promotes OL maturation. Thus, Tf could be used to enhance SVZ-derived progenitor proliferation and OL maturation when grafted into rat brains. Future endeavours will require a further understanding of the role played by different SVZ subpopulations during the oligodendrogenesis process in regards the molecular mechanisms and downstream events that underlie the Tf binding to its receptor. *In vivo* experiments will eventually become an unavoidable requisite to evaluate if the enhanced oligodendroglial maturation seen with aTf on SVZ-derived cells *in vitro*, renders a more robust myelinating capacity in a demyelination model.

## Supporting Information

Figure S1 **Non-dissociated neurosphere (NS) cultures.** Proliferating NS incorporate BrdU (A, red). Cell nuclei are shown concentrated in the center of the attached NS (B, white), with decreasing density gradient towards peripheral regions as cells migrate away from the NS. Cells close to the NS center express Nestin (C, green), some of which incorporate BrdU (red, whole arrowhead). Nestin^+^/BrdU^−^ cells are indicated with empty arrowheads in *C*. Most cells in proliferating NS express GFAP (D, green). Some MBP^+^ membranes (D, red) were found as well. BrdU incorporation is observed during *differentiation* (E, red), although GFAP expression is still observed in NS vicinity (F, green). MBP expression increases after *differentiation* (G, red) at a distance from the NS centre, and cells expressing the NG2 can still be detected (H, red). Differentiated GFAP^+^ (green) and MBP^+^ (red) cells are shown in *I*. Blue colour in images indicates Höechst nuclear dye. Scale bar in A equals 250 µm in A, E and F, scale bar in B equals 200 µm in B, G and H, and scale bar in C equals 10 µm in C, D and 5 µm in I.
(TIF)

Figure S2 **Tf effects on non-dissociated NS cultures.** MBP (red) is expressed in cells derived from attached NS (A–C). White outlined rectangles in A exemplify the image sections used to semi-quantitate MBP levels in four areas neighbouring each NS. The rectangles used for the representative images A, B and C are shown stacked-up and enlarged on the right hand side of their corresponding image. The MBP immunofluorescence was evaluated in terms of its Integrated Optical Density (IOD) in each rectangle and relativized to the number of nuclei present in it (D and G), as an estimate parameter of MBP protein expression. MBP^+^ pixel area was relativized to the total nuclei in each rectangle (E and F) to evaluate overall MBP^+^ cell process extension and surface coverage. Blue colour in images indicates Höechst nuclear dye. Scale bar in A represents 250 µm for A, B and C. Images 1–4 are twice the size as their corresponding insets in A. Bars in all graphs represent Mean + SEM of a single experiment. *** p<0.001. The analysis was performed from a single original culture.
(TIF)

Figure S3 **Neurosphere size relative frequencies.** A: The NS radius length of free floating NS is shown compared amongst the different conditions and at different time points. *B*: The radii of NS under different conditions were plotted as a frequency distribution with relative frequencies tabulated as percent values from a single culture. ApoTransferrin-treated cultures have a larger radius compared to controls. NS size heterogeneity increases with time. Data in A and B belong to at least 700 NS per condition. The statistical analysis in A was performed using a One Way ANOVA using the data of 100 randomly selected NE of each condition. * p<0.05, ** p<0.01, *** p<0.001.
(TIF)

Figure S4 **Tf and Transferrin Receptor (TfRc) Expression in SVZ explant cultures.** Subventricular zone tissue explants cultures show TfRc expression, mainly in cells within and close to the explant (A, green). Tf immunodetection (B, red) was observed in cells within the explants and in cells that migrated away from the explants center. Not all cells in this culture system co-express Tf (red) and TfRc (green) as shown in *C*. Inset in *C* is shown in *C′*. The blue colour in B indicates Höechst nuclear dye. Scale bar in A equals 200 µm in A–C, and scale bar in C′ equals 100 µm.
(TIF)
