# Supplementary figures and images for: ApoTransferrin: Dual Role on Adult Subventricular Zone-Derived Neurospheres
Source: PLoS One. 2012 Mar 30;7(3):e33937. doi: 10.1371/journal.pone.0033937 (PMC3316520; doi:10.1371/journal.pone.0033937)

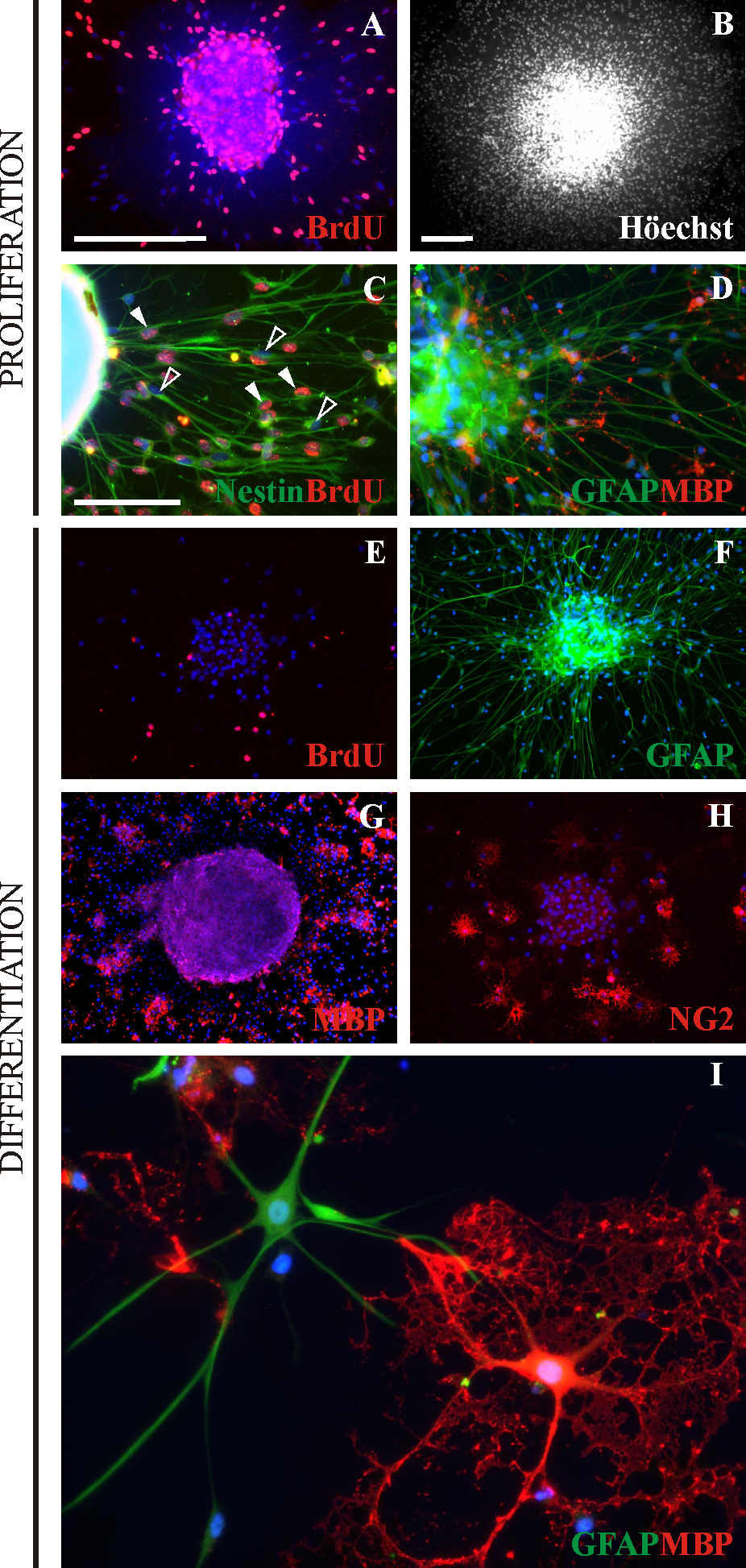

Supplement: Figure S1 — Non-dissociated neurosphere (NS) cultures. Proliferating NS incorporate BrdU (A, red). Cell nuclei are shown concentrated in the center of the attached NS (B, white), with decreasing density gradient towards peripheral regions as cells migrate away from the NS. Cells close to the NS center express Nestin (C, green), some of which incorporate BrdU (red, whole arrowhead). Nestin+/BrdU− cells are indicated with empty arrowheads in C. Most cells in proliferating NS express GFAP (D, green). Some MBP+ membranes (D, red) were found as well. BrdU incorporation is observed during differentiation (E, red), although GFAP expression is still observed in NS vicinity (F, green). MBP expression increases after differentiation (G, red) at a distance from the NS centre, and cells expressing the NG2 can still be detected (H, red). Differentiated GFAP+ (green) and MBP+ (red) cells are shown in I. Blue colour in images indicates Höechst nuclear dye. Scale bar in A equals 250 µm in A, E and F, scale bar in B equals 200 µm in B, G and H, and scale bar in C equals 10 µm in C, D and 5 µm in I. (TIF) [file pone.0033937.s001.tif]

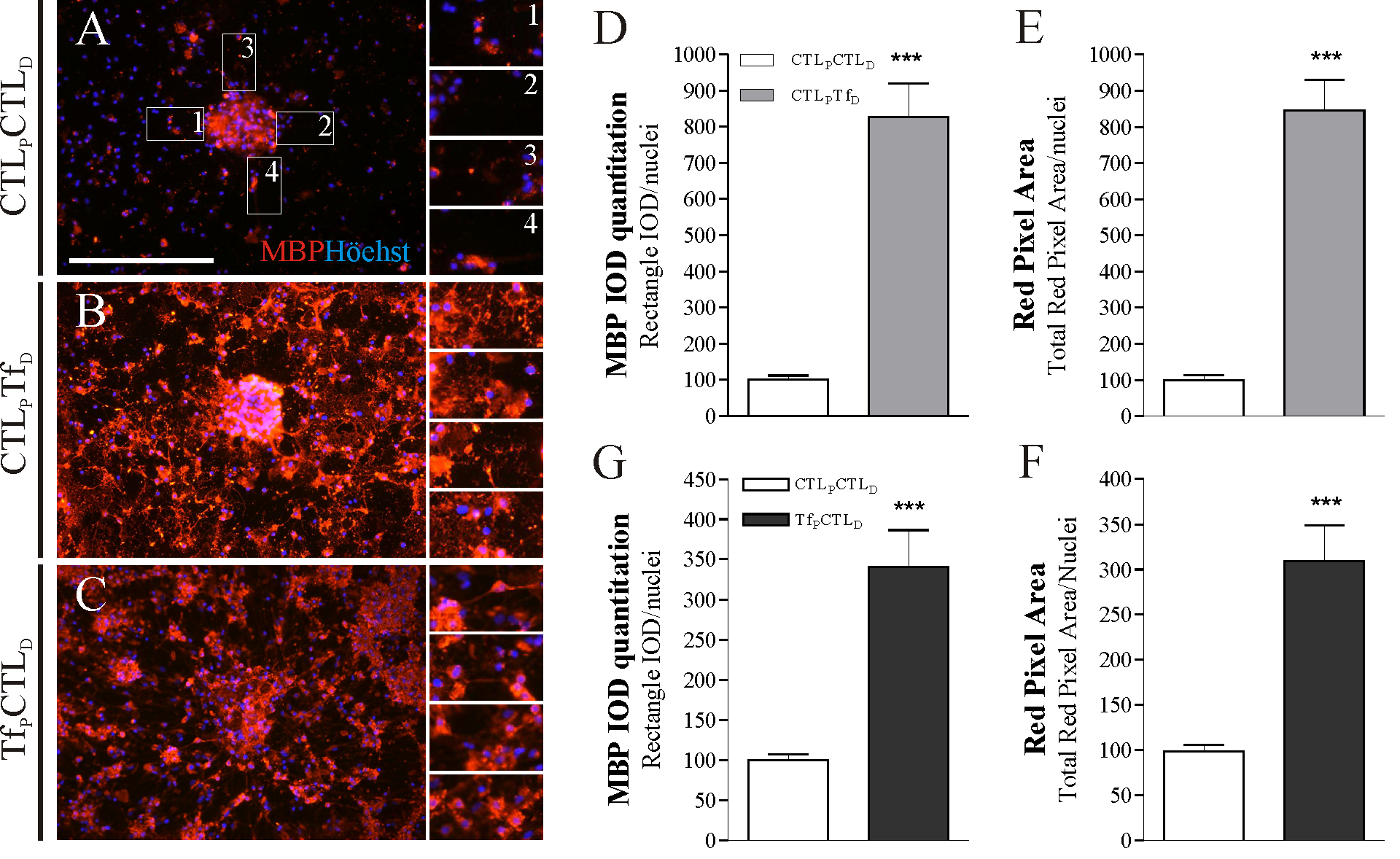

Supplement: Figure S2 — Tf effects on non-dissociated NS cultures. MBP (red) is expressed in cells derived from attached NS (A–C). White outlined rectangles in A exemplify the image sections used to semi-quantitate MBP levels in four areas neighbouring each NS. The rectangles used for the representative images A, B and C are shown stacked-up and enlarged on the right hand side of their corresponding image. The MBP immunofluorescence was evaluated in terms of its Integrated Optical Density (IOD) in each rectangle and relativized to the number of nuclei present in it (D and G), as an estimate parameter of MBP protein expression. MBP+ pixel area was relativized to the total nuclei in each rectangle (E and F) to evaluate overall MBP+ cell process extension and surface coverage. Blue colour in images indicates Höechst nuclear dye. Scale bar in A represents 250 µm for A, B and C. Images 1–4 are twice the size as their corresponding insets in A. Bars in all graphs represent Mean + SEM of a single experiment. *** p<0.001. The analysis was performed from a single original culture. (TIF) [file pone.0033937.s002.tif]

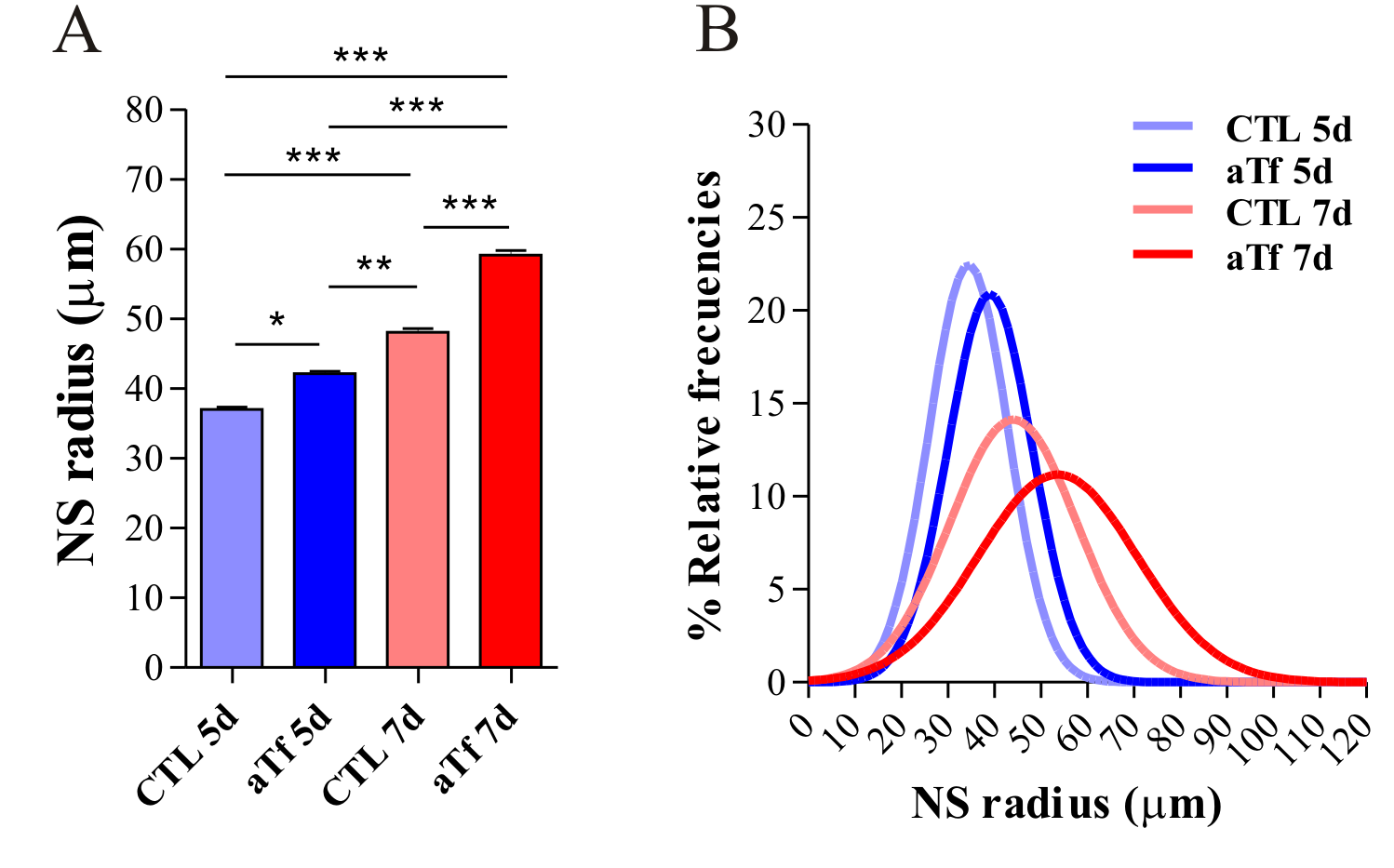

Supplement: Figure S3 — Neurosphere size relative frequencies. A: The NS radius length of free floating NS is shown compared amongst the different conditions and at different time points. B: The radii of NS under different conditions were plotted as a frequency distribution with relative frequencies tabulated as percent values from a single culture. ApoTransferrin-treated cultures have a larger radius compared to controls. NS size heterogeneity increases with time. Data in A and B belong to at least 700 NS per condition. The statistical analysis in A was performed using a One Way ANOVA using the data of 100 randomly selected NE of each condition. * p<0.05, ** p<0.01, *** p<0.001. (TIF) [file pone.0033937.s003.tif]

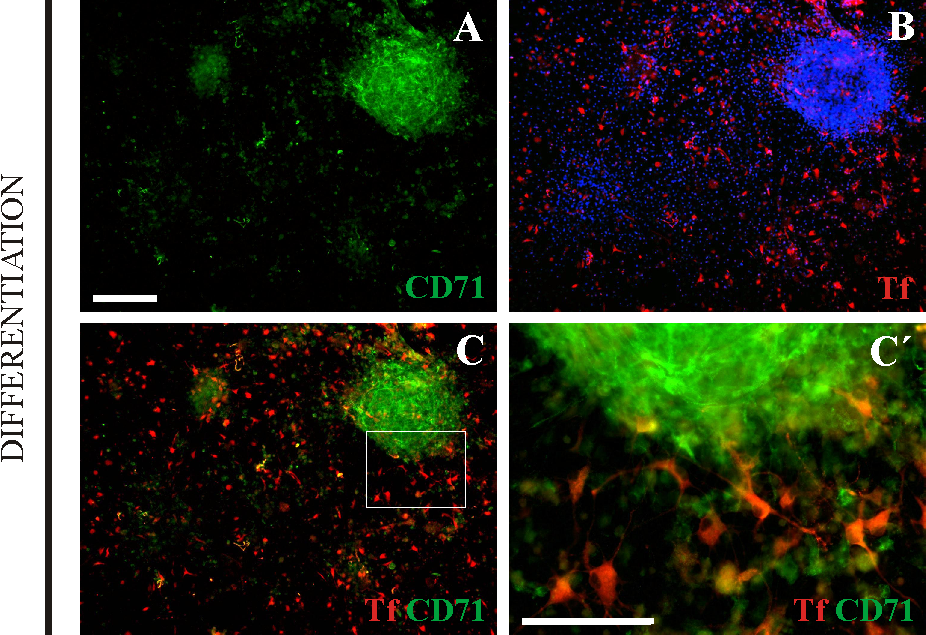

Supplement: Figure S4 — Tf and Transferrin Receptor (TfRc) Expression in SVZ explant cultures. Subventricular zone tissue explants cultures show TfRc expression, mainly in cells within and close to the explant (A, green). Tf immunodetection (B, red) was observed in cells within the explants and in cells that migrated away from the explants center. Not all cells in this culture system co-express Tf (red) and TfRc (green) as shown in C. Inset in C is shown in C′. The blue colour in B indicates Höechst nuclear dye. Scale bar in A equals 200 µm in A–C, and scale bar in C′ equals 100 µm. (TIF) [file pone.0033937.s004.tif]
